# Fitness, body composition, and metabolic risk scores in children and adolescents: the UP&DOWN study

**DOI:** 10.1007/s00431-022-04707-1

**Published:** 2022-11-29

**Authors:** Alejandro Sánchez-Delgado, Alejandro Pérez-Bey, Rocío Izquierdo-Gómez, José Jimenez-Iglesias, Ascensión Marcos, Sonia Gómez-Martínez, María José Girela-Rejón, Oscar L Veiga, José Castro-Piñero

**Affiliations:** 1 GALENO Research Group, Department of Physical Education, Faculty of Education Sciences, University of Cadiz, 11519 Puerto Real, Cádiz, Spain; 2 Instituto de Investigación e Innovación Biomédica de Cádiz (INiBICA), Cádiz, Spain; 3 Department of Metabolism and Nutrition (DMN) , Institute of Food Science and Technology and Nutrition (ICTAN), Spanish National Research Council (CSIC), Madrid, 28040 Spain; 4 Physical Activity for HEaLth Promotion research group (PA-HELP), Faculty of Education Sciences, Department of Didactic of Corporal Expression, University of Granada, Granada, Spain; 5 EstiLIFE Research Group, Department of Physical Education, Sports and Human Movement, Autonomous University of Madrid, Madrid, Spain

**Keywords:** Cardiovascular disease, Aerobic capacity, Muscular strength, Youth, Adiposity

## Abstract

We aimed to analyse the longitudinal association between physical fitness (PF) and body composition (BC) with a metabolic risk score (Met4) in children and adolescents and to elucidate whether the association between PF and Met4 differs when using relativized or absolute fitness variables. A total of 188 children (86 females) and 195 adolescents (97 females) were included. Cardiorespiratory fitness (CRF) was determined by the 20-m shuttle run test, and muscular fitness (MF) was determined by hand grip and standing long jump tests. Height and weight were measured, and the body mass index (Kg/m^2^) was calculated. Triceps and subscapular skinfolds were assessed to compute body fat percentage. Met4 was computed from systolic blood pressure, triglycerides, high-density lipoprotein cholesterol, and glucose levels. Relative CRF was longitudinally and negatively associated with Met4 in female children (*β* =  −0.031, *p* = 0.025), while absolute CRF was positively associated with Met4 in male children and adolescents (*β* = 0.000, *p* < 0.05). Relative upper and lower-body MF were longitudinally and negatively associated with Met4 in female adolescents (*β* =  −1.347, β =  −0.005, *p* < 0.05), while absolute lower-body MF was positively associated with Met4 in male children (*β* = 0.000, *p* = 0.019). BC was longitudinally and positively associated with Met4 in male children (β-ranging from 0.011 to 0.055, all *p* < 0.05) and male adolescents (β-ranging from 0.011 to 0.046, all p < 0.05).

*  Conclusion*: BC is more strongly associated with Met4 than PF in children and adolescents. An optimal body weight status should be considered the main objective of health-promoting programs at childhood and adolescence. Furthermore, the way of expressing the fitness variables determines the direction of the association with Met4.

**Table:** 

**What is Known:** *• Physical fitness is an important health indicator in children and adolescents, with great amount of previous evidence supporting the preventive role of maintaining optimal levels of both cardiorespiratory and muscular fitness for future cardiometabolic issues.*	
**What is New:** *• The way of reporting physical fitness variables can affect the associations between physical fitness features and cardiometabolic outcomes. Since body composition variables have a great impact on both physical fitness and cardiometabolic health, relativizing physical fitness performance by body composition could lead to erroneous conclusions.*

**What is Known:**

*• Physical fitness is an important health indicator in children and adolescents, with great amount of previous evidence supporting the preventive role of maintaining optimal levels of both cardiorespiratory and muscular fitness for future cardiometabolic issues.*

**What is New:**

*• The way of reporting physical fitness variables can affect the associations between physical fitness features and cardiometabolic outcomes. Since body composition variables have a great impact on both physical fitness and cardiometabolic health, relativizing physical fitness performance by body composition could lead to erroneous conclusions.*

## Introduction

Cardiovascular diseases (CVD) are one of the leading causes of global mortality [1]. In 2015, CVD was responsible for 17.9 million deaths, and unfortunately, an increase in CVD mortality has been projected due to population aging and growth [1]. Although CVD occurs mainly after the 5th decade of life [2], its precursors are already present at early ages [3]. Interestingly, the cluster of CVD risk factor levels in childhood and adolescence track into adulthood [4, 5]. The well-known metabolic syndrome score (MetS), which is the clustering of abdominal obesity, elevated systolic blood pressure (SBP), elevated triglycerides, decreased high-density lipoprotein cholesterol (HDL-C), and elevated fasting plasma glucose, remains a major public health burden with the prevalence of the syndrome increasing in concert with obesity and sedentary lifestyles [6]. MetS affects both youths and adults and has been linked with clinical manifestations in CVD and type 2 diabetes [7, 8].

Physical fitness (PF) is an important health marker in youth [9, 10]. Specifically, having higher levels of cardiorespiratory fitness (CRF) [11, 12] and muscular fitness (MF) [9, 13] at an early age is associated with lower levels of mortality and lower levels of CVD risk factors. On the other hand, adiposity is another fundamental aspect that has been strongly related with cardiovascular health, so that high levels of adiposity in youth are accompanied by a worse CVD profile [14, 15], and this outcome persists later in life [10, 16].

An essential aspect is that PF and adiposity are closely related [17], what makes it difficult to elucidate from longitudinal observational studies whether PF, adiposity, or both are predictive of CVD risk. This problem is derived from the causal relationship between these both features, since this relationship becomes more difficult to solve when it treats with PF measures in absolute terms and/or relativized by anthropometric measures as equals [18]. In terms of MF, a recent systematic review and meta-analysis showed how the association between MF and several health outcomes can change and even reverse if relative MF (i.e., divided by body mass) or absolute MF are used [19].

Recent systematic reviews showed that most of the studies including PF measures use their relativized form [18, 19], which could lead to misconceptions of the independent effect that PF exerts on the outcome, given the interferences produced by adiposity. However, to our knowledge, few studies included PF with in its absolute form [20, 21]. Furthermore, most of the previous evidence comes from cross-sectional studies [18], being more longitudinal designs needed, not only to assess the longitudinal association but also to analyse how the progression of the exposures of interest affect the progression of the MetS through the years. This knowledge would help clarify the role of PF and body composition (BC), as well as their interaction, on MetS in children and adolescents. This in practice would bring to greater knowledge on how to evaluate and express PF variables at both, the school setting and in future research where current and future MetS is considered the outcome in children and adolescents. Given this, we aimed to analyse the longitudinal association between PF (i.e., MF and CRF) and BC with MetS in children and adolescents and to elucidate whether the association between PF and MetS differs when using relativized or absolute PF variables.

## Materials and methods

### Study design and population

Participants took part of the UP&DOWN study [22]. In brief, this 2-year longitudinal study aimed to assess the impact on health indicators of physical activity, sedentary behaviours, and health-related PF in apparently healthy primary and secondary schoolchildren from Spain. The total UP&DOWN study sample consisted of 2264 healthy children (6–11.9 years) and adolescents (12–17.9 years) enrolled from schools in Cádiz and Madrid, respectively, of whom 1226 were children (580 females) and 1038 adolescents (502 females). According to the Spanish Institute of National Statistics, the UP&DOWN sample size represents 50% and 5% of the total population of schoolchildren and adolescents, respectively. After selecting a random one-fourth for blood sampling, the resulting sample with complete data of PF, BC, SBP, and blood sampling at baseline was 461 children and adolescents, being 231 children (108 females) and 230 adolescents (111 females). In the follow-up, some participants dropped out of the study (18.6% of children and 15.2% of adolescents), and the final sample with complete data was 383 participants, of which 188 were children (86 females) and 195 adolescents (97 females). We collected baseline data from September 2011 to June 2012, and the follow-up was performed from September 2013 to June 2014. Participants’ parents were informed about the purposes of the study, and written informed consents were provided. The study protocol was accepted by the Ethics Committee of the Hospital Puerta del Hierro (Madrid, Spain), the Bioethics Committee of the National Research Council (Madrid, Spain), and the Committee for Research Involving Human Subjects of the University of Cádiz (Cádiz, Spain).

### Tanner stage

Explicative drawings of breast and genial development for females and males, respectively, were given to participants for self-classification in one of the five stages of pubertal development according to Tanner and Whitehouse [23].

### Systolic blood pressure

Systolic blood pressure was measured with a validated digital automatic blood pressure monitor (OMRON M6; OMRON HEALTH CARE Co., Ltd., Kyoto, Japan) according to the standardized and valid International Protocol of the European Society of Hypertension [24]. Two measurements were taken 1 to 2 min apart. If the first two readings differed in > 5 mm Hg, an additional measurement was taken, and the farthest value was removed. The average value of the two measurements was selected.

### Blood sampling

After an overnight fast, 13.5 mL of blood was extracted from the cubital vein of each participant. Once the blood was collected, it was immediately transported to standard laboratories in each city, using the same protocols. About 3.5 mL of the blood sample was collected in ethylenediaminetetraacetic acid (EDTA) and analysed to acquire hemogram data. The remaining blood was collected in dried gel and sodium citrate and centrifuged to remove serum and plasma. Finally, the serum was frozen at −80 °C for future analyses. In the current study, enzymatic colourimetric methods (Olympus AU2700 Analyzer; Olympus UK Ltd., Watford, UK) were used to analyse serum lipid triglycerides, high-density lipoprotein cholesterol (HDL-c), and glucose.

### Body composition

All BC variables were obtained in the morning between 9:00 a.m. and 12:00 p.m., just before performing PF tests. Weight and height were measured with an electronic scale (type SECA 861; range, 0.05–130 kg; precision, 0.05 kg; Hamburg, Germany) and a telescopic stature-measuring instrument (type SECA 225; range, 60–200 cm; precision, 1 mm; Hamburg, Germany), respectively. These measurements were conducted with participants dressed in lightweight clothing and without shoes. Body mass index (BMI) was calculated as weight/height squared (kg/m^2^). Waist circumference (WC) was measured at the level of the narrowest part of the torso, using a non-elastic tape (SECA 200; range, from 0 to 150 cm; precision, 1 mm; Hamburg, Germany). Triceps and subscapular skinfolds were assessed according to Lohman’s anthropometric standardization reference manual [25] by trained professionals. Skinfolds were taken on the non-dominant side of the body using a Holtain skinfold calliper (range, from 0 to 40 mm; precision, 0.2 mm), and body fat percentage (%BF) was estimated with Slaughter equations [26]. Two non-consecutive measurements of all BC variables were carried out, and the average was recorded.

### Cardiorespiratory fitness

The 20-m shuttle run test was used to assess CRF [27]. Participants were asked to run between two lines 20 m apart at a pace marked by a prerecorded sound signal. The initial speed was 8.5 km/h, with 0.5 km/h increments each stage. The test finished when the participant could not reach the line twice in a row. The last half-stage attained was recorded and used to estimate relative maximal oxygen uptake (VO_2_max [mL/kg/min]) through Léger’s equation [27]. This test has shown to be valid and reliable in children and adolescents [28, 29]. Finally, we obtained absolute VO_2_max was obtained by multiplying relative VO_2_max by body weight.

### Muscular fitness

Upper and lower body strength was assessed by the handgrip strength (HG) and standing long jump tests, respectively. The HG test was conducted using a validated hand dynamometer with an adjustable grip (TKK 5101 Grip D; Takey, Tokyo, Japan) [30]. After adjusting the grip span to the hand size according to the equations specifically developed for children [31] and adolescents [32], participants were asked to squeeze gradually and continuously for at least 2 s. The test was performed twice, and the maximum score for each hand was recorded in kilograms. The average score of the left and right hands was used as the absolute upper MF. Furthermore, to obtain relative upper MF, absolute upper MF was divided by body weight (HG/weight) to preclude body size influences [33]. The standing long jump test (SLJ) was performed with participants standing behind a line with feet approximately shoulder’s width apart. From this position, participants jumped as far forwards as possible. The test was performed twice, and the best score was recorded in centimetres. This score was used as a relative lower MF, and the product of centimetres obtained per body weight was used as an absolute lower MF. A single muscular fitness score (i.e., global MF) was calculated as a standardized score by age groups (i.e., children and adolescents) and sex (i.e., male and female) composed of relative MF of the upper and lower body [34].

### Overall fitness

An overall fitness score was computed as the mean of the global MF and the relative CRF values (paliers) standardized by age groups and by sex.

### Metabolic risk scores

A MetS was created from the mean of the standardized values of each individual CVD risk factor (i.e., WC, SBP, triglycerides, HDL-c, and glucose) by age groups (children and adolescents) and sex (males and females). This index has been previously used by the International Diabetes Federation to assess cardiovascular health in children and adolescents [35].

The standardized value for HDL-c was multiplied by (−1) since higher HDL-c levels represent lower CVD risk. Given the close relationship between BC variables and WC. Additionally, a different metabolic risk score was computed excluding WC (i.e., Met4), given the close relationships between WC and the rest of the BC measures used as independent variables [36–38].

### Data analyses

Significant interactions by sex (males and females) and age groups (children and adolescents) in the studied associations were observed. Consequently, all analyses were performed by sex and age groups. Descriptive statistics are presented as mean ± standard deviation. T-tests were used to check differences in variables of interest between sex for both age groups at both time points. All variables were checked for normality.

To examine the cross-sectional association of PF and BC variables with MetS scores, we used linear regression models, where PF variables (i.e., 20-m shuttle run test, relative VO_2_max (ml/kg/min), absolute VO_2_max (ml/min), absolute upper MF, relative upper MF, absolute lower MF, relative lower MF, global MF, overall fitness), and BC variables (i.e., body weight, BMI, fat mass percentage, and WC) at baseline were individually introduced as independent variables, and MetS and Met4 scores at baseline were individually introduced as dependent variables. All analyses were controlled for age, educational centre, and the mother’s education level at baseline.

To study the longitudinal association between PF variables and Met4, we used linear regression models where PF variables at baseline were individually introduced as independent variables and Met4 at 2-years follow-up as the dependent variable. In model 1, we adjusted by age, educational centre, mother’s education level, and Met4 at baseline; model 2 was model 1 + WC at baseline; and model 3 was model 1 + %BF at baseline.

To examine the longitudinal association between BC variables and Met4, linear regression models were used, where BC variables (i.e., weight, BMI, %BF, and WC) at baseline were individually introduced as independent variables and Met4 at 2-years follow-up as the dependent variable. In model 1, we adjusted by age, educational centre, mother’s education level and Met4 at baseline, model 2 was model 1 + relative CRF (paliers) at baseline, model 3 was model 1 + global MF at baseline and model 4 was model 1 + overall fitness score at baseline.

To analyse whether changes in PF variables are associated with future Met4, linear regression models were used, where the change (follow-up value—baseline value) of 20-m shuttle run test, relative VO2max, absolute VO_2_max, absolute upper MF, relative upper MF, absolute lower MF, relative lower MF, global MF, and overall fitness score were individually introduced as independent variables, and Met4 at 2-years follow-up were individually introduced as the dependent variable. All models were adjusted by age, educational centre, mother’s education level, and Met4 at baseline. In addition, changes in PF variables were adjusted by changes in WC, while changes in PF variables were adjusted by changes in %BF. The same analyses were performed but introducing the changes in Met4 as the dependent variable in order to test whether changes in PF variables were associated with changes in Met4. Analyses were controlled for the same variables except for Met4 at baseline.

To study whether changes in BC variables are associated with future Met4, linear regression models were used, where the change of weight, BMI, %BF, and WC were individually introduced as independent variables and Met4 at 2-years follow-up was individually introduced as the dependent variable. All models were adjusted by age, educational centre, mother’s education level, and Met4 at baseline. Moreover, changes in PF variables (CRF, MF, and overall fitness) were used individually as adjustment variables. The same analyses were performed but introducing the changes in Met4 as the dependent variable in order to test whether changes in BC variables were associated with changes in Met4. Analyses were controlled for the same variables mentioned above except Met4 at baseline.

Finally, to evaluate how changes in BC variables are associated with Met4 at follow-up, cut points for WC [39], %BF [40], and BMI were used to create the different BC groups at baseline and follow-up. WC and %BF groups were created as dichotomic variables (0 without risk, 1 with risk) in the function of sex and age, and BMI was created as a dichotomic variable, where 0 (without risk) was assigned to underweight and normal weight and 1 (with risk) was assigned to overweight and obesity. Residuals of the model in which Met4 at follow-up was a dependent variable, changes in overall fitness were considered the independent variable and age, educational centre, mother’s education level, and Met4 at baseline were adjustment variables, were used as dependent variable and changes in BC groups were used as independent variable. Analyses were performed using the environment for statistical computing R [41], version 4.0.3 (R Foundation for Statistical Computing, Vienna, Austria). The significance was set at *P* < 0.05.

## Results

Participant characteristics are shown in Table 1. Overall, at baseline, female children and adolescents displayed higher levels in the sum of skinfolds and %BF, and lower levels of CRF and MF variables (all *p* < 0.05) compared to male children and adolescents, except in absolute upper- and lower-body MF in children. At follow-up, female children had higher levels of %BF and lower levels of CRF and relative lower-body MF (all *p* < 0.05) than males. In adolescents, females presented lower SBP, glucose levels, CRF and MF, higher HDL-c, and higher body fatness indicators levels compared to males (all *p* < 0.05).

**Table 1:** Baseline and 2-y follow-up characteristics of the study sample by age and sex

**Baseline**						
	**Male children**	**Female children**	***P* value**	**Male adolescents**	**Female adolescents**	***P* value**
	(*n* = 123)	(*n* = 108)		*(n* = 119)	(*n* = 111)	
Tanner stage	1.60 (0.63)	1.44 (0.64)	0.096	3.52 (0.91)	3.41 (0.76)	0.370
Age (years)	8.12 (1.51)	8.17 (1.49)	0.812	13.98 (1.60)	13.84 (1.44)	0.536
Systolic blood pressure (mmHg)	101.85 (11.14)	99.95 (10.70)	0.238	109.79 (13.13)	106.71 (9.46)	0.062
Triglycerides (mg/dL)	40.07 (19.07)	44.98 (16.68)	0.064	48.45 (20.25)	52.26 (20.66)	0.195
HDL Cholesterol (mg/dL)	39.71 (16.46)	40.88 (16.43)	0.625	48.48 (15.04)	50.16 (14.73)	0.430
Glucose (mg/dL)	60.91 (17.96)	62.02 (16.86)	0.664	79.63 (16.29)	77.80 (15.39)	0.421
*Body composition*						
Weight (kg)	30.72 (8.23)	31.38 (10.30)	0.624	53.43 (12.02)	51.90 (8.99)	0.315
Height (cm)	129.33 (10.06)	130.29 (11.58)	0.544	161.62 (11.67)	157.88 (6.32)	**0.006**
Body mass index (kg/m^2^)	18.08 (2.81)	18.05 (3.55)	0.949	20.24 (2.88)	20.76 (2.96)	0.223
Waist circumference (cm)	59.30 (6.94)	58.15 (8.01)	0.293	68.64 (6.94)	65.83 (5.65)	**0.002**
Sum of two skinfolds (mm)	21.72 (9.69)	25.04 (12.59)	**0.043**	22.25 (11.89)	28.78 (10.59)	** < 0.001**
Body fat (%)	22.13 (9.05)	25.77 (8.92)	**0.006**	21.10 (10.47)	29.92 (7.17)	** < 0.001**
*Cardiorespiratory fitness*						
20-m shuttle run test (paliers)	3.28 (1.74)	2.48 (1.73)	**0.002**	7.03 (2.55)	4.41 (1.87)	** < 0.001**
Relative VO_2_max (ml/kg/min)	47.96 (3.93)	46.05 (4.08)	**0.001**	47.81 (5.73)	40.89 (4.72)	** < 0.001**
Absolute VO_2_max (ml/min)	1456.60 (330.48)	﻿1424.28 (406.26)	0.548	2554.45 (656.50)	2114.80 (412.24)	** < 0.001**
*Muscular fitness (MF)*						
Absolute upper-body MF (kg)	12.46 (3.62)	11.65 (3.29)	0.112	26.63 (8.54)	22.54 (4.82)	** < 0.001**
Relative upper-body MF (kg/weight)	0.41 (0.07)	0.38 (0.08)	**0.029**	0.50 (0.10)	0.44 (0.08)	** < 0.001**
Relative lower-body MF (cm)	115.68 (21.03)	109.14 (22.67)	**0.042**	174.62 (33.80)	142.55 (20.95)	** < 0.001**
Absolute lower-body MF (cm*weight)	3557.92 (1127.79)	3415.93 (1238.50)	0.412	9497.27 (3377.14)	7425.93 (1788.94)	** < 0.001**
**Follow-up**						
	**Male children**	**Female children**	***P* value**	**Male adolescents**	**Female adolescents**	***P* value**
	(*n* = 102)	(*n* = 86)		*(n* = 98)	(*n* = 97)	
Tanner stage	2.31 (0.61)	2.17 (1.04)	0.257	4.44 (0.63)	3.98 (0.61)	** < 0.001**
Age (years)	10.16 (1.49)	10.22 (1.50)	0.764	15.98 (1.59)	15.84 (1.44)	0.518
Systolic blood pressure (mmHg)	105.29 (9.17)	105.28 (10.72)	0.995	113.18 (12.21)	103.54 (9.50)	** < 0.001**
Triglycerides (mg/dL)	41.83 (19.61)	46.50 (25.18)	0.155	63.49 (25.88)	62.23 (22.99)	0.719
HDL Cholesterol (mg/dL)	39.49 (14.93)	40.55 (16.78)	0.648	52.59 (12.48)	59.39 (11.84)	** < 0.001**
Glucose (mg/dL)	71.24 (16.68)	73.16 (17.35)	0.439	87.04 (7.93)	84.73 (8.21)	**0.047**
*Body composition*						
Weight (kg)	38.79 (10.59)	40.00 (13.06)	0.484	62.47 (10.88)	56.28 (8.80)	** < 0.001**
Height (cm)	140.79 (10.15)	143.25 (12.13)	0.132	171.69 (7.87)	162.19 (5.18)	** < 0.001**
Body mass index (kg/m^2^)	19.28 (3.39)	19.04 (3.98)	0.662	21.11 (2.93)	21.40 (3.23)	0.510
Waist circumference (cm)	63.51 (8.50)	61.23 (9.07)	0.077	71.78 (6.67)	67.11 (6.58)	** < 0.001**
Sum of two skinfolds (mm)	26.81 (13.72)	28.46 (14.59)	0.424	19.96 (9.56)	29.50 (12.71)	** < 0.001**
Body fat (%)	24.87 (10.65)	27.84 (8.85)	**0.041**	18.40 (9.06)	30.18 (7.59)	** < 0.001**
*Cardiorespiratory fitness*						
20-m shuttle run test (paliers)	4.45 (1.81)	3.36 (1.99)	** < 0.001**	8.19 (2.19)	4.65 (1.87)	** < 0.001**
Relative VO_2_max (ml/kg/min)	47.12 (4.65)	44.46 (4.46)	** < 0.001**	48.03 (6.25)	38.14 (5.82)	** < 0.001**
Absolute VO_2_max (ml/min)	1797.63 (391.28)	1754.90 (503.30)	0.514	2985.39 (572.84)	2134.77 (411.20)	** < 0.001**
*Muscular fitness (MF)*						
Absolute upper-body MF (kg)	15.55 (3.81)	15.77 (4.67)	0.716	34.85 (7.44)	26.06 (3.93)	** < 0.001**
Relative upper-body MF (kg/weight)	0.41 (0.08)	0.41 (0.09)	0.738	0.56 (0.09)	0.47 (0.08)	** < 0.001**
Relative lower-body MF (cm)	131.65 (22.93)	124.72 (24.68)	**0.048**	194.50 (29.83)	146.78 (21.56)	** < 0.001**
Absolute lower-body MF (cm*weight)	5094.80 (1596.12)	4983.33 (1812.90)	0.654	12,213.52 (2994.01)	8244.00 (1676.72)	** < 0.001**

Values are presented as mean (standard deviation). Statistically significant differences between sexes in variables are highlighted in bold

*HDL *high density lipoprotein, *VO*_*2*_*max *maximum oxygen consumption

Cross-sectional associations between PF and BC variables with MetS scores at baseline are depicted in Table 2. Relative CRF (i.e., paliers and relative VO_2_max) was negatively associated with Met4 in male children and in adolescents of both sexes (β ranging from −0.024 to −0.082, all *p* < 0.05). Besides, absolute CRF (i.e., absolute VO_2_max) was positively associated with MetS scores in children and adolescents of both sexes, although the effect size was small (β ranging from 0.0 to 0.001, all *p* < 0.05). Absolute upper- and lower-body MF were positively associated with Met4 in female children (*β* = 0.072 and *β* = 0.000, respectively, both *p* < 0.001). In male adolescents, relative upper-body MF was negatively associated with Met4 (*β* =  −1.784, *p* = 0.001), while absolute lower-body MF was positively associated with Met4 (*β* = 0.000, *p* = 0.001). Global MF and overall fitness score were negatively associated with MetS in both sexes and all age groups (β ranging from −0.195 to −0.419, all *p* < 0.05). These scores were negatively associated with Met4 in male children and adolescents (β ranging from −0.173 to −0.279, all *p* < 0.05). All BC variables were positively associated with Met4 in both sexes and all age groups (β ranging from 0.012 to 0.112, all *p* < 0.05). The same results were found when controlled by the tanner stage at baseline instead of age.

**Table 2:** Cross-sectional association between fitness and body composition indicators with metabolic risk scores in children and adolescents

	**Children (*n* = 231)**													
	**Male (*n* = 123)**							**Female (*n* = 108)**						
	MetS			Met4			MetS					Met4		
	Adjusted R^2^	*β*	*p*	Adjusted R^2^	*β*	*p*	Adjusted R^2^			*β*	*p*	Adjusted R^2^	*β*	*p*
*Cardiorespiratory fitness*														
20-m shuttle run test	0.270	−0.112	** < 0.001**	0.155	−0.082	**0.002**	0.119			−0.057	0.134	0.062	−0.014	0.682
Relative VO_2_max	0.272	−0.050	** < 0.001**	0.154	−0.036	**0.002**	0.118			−0.024	0.144	0.061	−0.006	0.694
Absolute VO_2_max	0.474	0.001	** < 0.001**	0.175	0.001	**0.001**	0.607			0.001	** < 0.001**	0.347	0.001	** < 0.001**
*Muscular fitness (MF)*														
Absolute upper-body MF	0.214	0.054	**0.002**	0.102	0.034	0.038	0.337			0.094	** < 0.001**	0.243	0.072	** < 0.001**
Relative upper-body MF	0.258	−2.244	** < 0.001**	0.098	−1.130	0.046	0.170			−1.628	**0.012**	0.079	−0.719	0.228
Relative lower-body MF	0.226	−0.007	**0.001**	0.097	−0.004	0.051	0.100			−0.003	0.380	0.059	0.000	0.933
Absolute lower-body MF	0.258	0.000	** < 0.001**	0.109	0.000	0.025	0.437			0.000	** < 0.001**	0.273	0.000	** < 0.001**
*Overall*														
Global MF	0.282	−0.328	** < 0.001**	0.111	−0.173	**0.023**	0.150			−0.205	**0.031**	0.070	−0.078	0.371
Overall fitness	0.314	−0.398	** < 0.001**	0.151	−0.246	**0.003**	0.145			−0.227	**0.039**	0.067	−0.074	0.460
*Body composition*														
Weight	0.600	0.048	** < 0.001**	0.246	0.027	** < 0.001**	0.597			0.042	** < 0.001**	0.320	0.027	** < 0.001**
Body mass index	0.567	0.110	** < 0.001**	0.215	0.059	** < 0.001**	0.638			0.106	** < 0.001**	0.347	0.068	** < 0.001**
Body fat percentage	0.377	0.024	** < 0.001**	0.127	0.012	**0.010**	0.364			0.032	** < 0.001**	0.184	0.019	**0.002**
Waist circumference	-	-	-	0.215	0.025	** < 0.001**	-			-	-	0.347	0.031	** < 0.001**
	**Adolescents (*n* = 130)**													
	**Male (*n* = 119)**								**Female (n = 111)**					
	MetS			Met4			MetS					Met4		
	Adjusted R^2^	*β*	*p*	Adjusted R^2^	*β*	*p*	Adjusted R^2^			*β*	*p*	Adjusted R^2^	*β*	*p*
*Cardiorespiratory fitness*														
20-m shuttle run test	0.456	−0.108	** < 0.001**	0.416	−0.076	**0.001**	0.100			−0.072	**0.010**	0.159	−0.066	**0.013**
Relative VO_2_max	0.450	−0.040	** < 0.001**	0.411	−0.028	**0.001**	0.099			−0.026	**0.011**	0.159	−0.024	**0.013**
Absolute VO_2_max	0.472	0.001	** < 0.001**	0.400	0.000	**0.003**	0.149			0.000	**0.001**	0.093	0.000	0.587
*Muscular fitness (MF)*														
Absolute upper-body MF	0.349	0.027	**0.017**	0.348	0.016	0.115	0.097			0.033	**0.012**	0.101	0.013	0.325
Relative upper-body MF	0.473	−2.724	** < 0.001**	0.414	−1.784	**0.001**	0.113			−1.950	**0.005**	0.107	−0.843	0.218
Relative lower-body MF	0.331	−0.004	0.058	0.336	−0.002	0.286	0.020			0.000	0.893	0.090	0.001	0.826
Absolute lower-body MF	0.476	0.000	** < 0.001**	0.412	0.000	**0.001**	0.208			0.000	** < 0.001**	0.125	0.000	0.078
*Overall*														
Global MF	0.428	−0.313	** < 0.001**	0.385	−0.195	**0.008**	0.068			−0.195	**0.049**	0.096	−0.070	0.468
Overall fitness	0.498	−0.419	** < 0.001**	0.430	−0.279	** < 0.001**	0.106			−0.259	**0.008**	0.134	−0.185	**0.049**
*Body composition*														
Weight	0.675	0.036	** < 0.001**	0.495	0.022	** < 0.001**	0.418			0.034	** < 0.001**	0.162	0.014	**0.011**
Body mass index	0.672	0.112	** < 0.001**	0.480	0.067	** < 0.001**	0.384			0.093	** < 0.001**	0.148	0.037	**0.024**
Body fat percentage	0.661	0.031	** < 0.001**	0.502	0.020	** < 0.001**	0.237			0.029	** < 0.001**	0.145	0.015	**0.027**
Waist circumference	-	-	-	0.490	0.031	** < 0.001**	-			-	-	0.169	0.022	**0.008**

Analyses were controlled by age, educational centre and mother’s education level. Statistically significant values are highlighted in bold

*MetS *metabolic syndrome score, *Met4 *metabolic risk score excluding waist circumference, *VO*_*2*_*max *maximum oxygen consumption, *β *standardized coefficient

Table 3 shows longitudinal associations between PF variables at baseline and Met4 at a 2-years follow-up. Relative CRF expressed in paliers and VO_2_max were negatively associated with Met4 in female children (*β* =  −0.073, *β* =  −0.031, *p* = 0.025, respectively). Contrary, absolute CRF was positively associated with Met4 in male children and adolescents. Similar results were observed when %BF at baseline was included in the model (Model 3) (*β* =  −0.106, *β* =  −0.046, *p* = 0.008, respectively). Overall, PF was negatively associated with Met4 when analyses were adjusted by %BF (model 3) (*β* =  −0.283, *p* = 0.025). In female, adolescents’ relative upper- and lower-body MF, the global MF, and overall PF were negatively associated with Met4 (β ranging from −0.005 to −1.347, all *p* < 0.05). Moreover, the global MF was negatively associated with Met4 in model 2 and 3 (*β* =  −0.216, *p* = 0.03, *β* =  −0.23, *p* = 0.035, respectively), and absolute upper-body MF was negatively associated with Met4 (*β* =  −0.029, *p* = 0.036), and absolute lower-body MF was positively associated with Met4 (*β* = 0.000, *p* = 0.016) in model 2. Finally, in male children and adolescents not associations were found between PF variables and Met4 score (*p* > 0.05), except in male children where absolute lower-body MF was positively associated with Met4 in model 1 (*β* = 0.000, *p* = 0.019).

**Table 3:** Longitudinal association between fitness indicators with Met4 in children and adolescents

	**Children (*n* = 188)**							**Adolescents (*n* = 195)**							
	**Male (*n* = 102)**			**Female (*n* = 86)**			**Male (*n* = 98)**				**Female (*n* = 97)**				
	Adjusted R^2^	*β*	*p*	Adjusted R^2^	*β*	*p*	Adjusted R^2^		*β*	*p*	Adjusted R^2^	*β*			*p*
***Model 1***															
*Cardiorespiratory fitness*															
20-m shuttle run test	0.279	−0.029	0.328	0.392	−0.073	**0.025**	0.353		0.005	0.819	0.353	−0.035			0.212
Relative VO_2_max	0.281	−0.014	0.277	0.392	−0.031	**0.025**	0.353		0.002	0.793	0.353	−0.013			0.212
Absolute VO_2_max	0.309	0.000	**0.035**	0.349	0.000	0.462	0.390		0.000	**0.032**	0.340	0.000			0.820
*Muscular fitness (MF)*															
Absolute upper-body MF	0.280	0.018	0.304	0.345	−0.007	0.738	0.367		0.013	0.190	0.355	−0.017			0.182
Relative upper-body MF	0.293	−0.963	0.104	0.366	−0.868	0.129	0.359		−0.480	0.395	0.373	−1.347			**0.048**
Relative lower-body MF	0.271	0.000	0.853	0.345	0.001	0.827	0.375		−0.003	0.101	0.375	−0.005			**0.040**
Absolute lower-body MF	0.318	0.000	**0.019**	0.353	0.000	0.332	0.355		0.000	0.614	0.349	0.000			0.289
*Overall*															
Global MF	0.279	−0.080	0.323	0.353	−0.082	0.327	0.368		−0.100	0.174	0.391	−0.235			**0.012**
Overall fitness	0.281	−0.100	0.267	0.374	−0.169	0.076	0.356		−0.050	0.545	0.375	−0.197			**0.039**
***Model 2***															
*Cardiorespiratory fitness*															
20-m shuttle run test	0.313	−0.008	0.783	0.385	−0.065	0.066	0.374		0.024	0.343	0.357	−0.032		0.254	
Relative VO_2_max	0.314	−0.005	0.714	0.385	−0.028	0.065	0.375		0.009	0.324	0.358	−0.012		0.252	
Absolute VO_2_max	0.313	0.000	0.841	0.358	0.000	0.459	0.385		0.000	0.136	0.370	0.000		0.096	
*Muscular fitness (MF)*															
Absolute upper-body MF	0.313	0.005	0.762	0.360	−0.019	0.383	0.373		0.009	0.368	0.383	−0.029		**0.036**	
Relative upper-body MF	0.315	−0.321	0.634	0.362	−0.640	0.329	0.367		−0.052	0.935	0.367	−1.170		0.119	
Relative lower-body MF	0.326	0.003	0.207	0.357	0.002	0.520	0.379		−0.003	0.214	0.379	−0.005		0.050	
Absolute lower-body MF	0.330	0.000	0.152	0.353	0.000	0.819	0.368		0.000	0.732	0.394	0.000		**0.016**	
*Overall*															
Global MF	0.314	0.031	0.735	0.354	−0.036	0.699	0.371		−0.058	0.476	0.386	−0.216		**0.030**	
Overall fitness	0.313	0.001	0.991	0.368	−0.138	0.205	0.367		0.023	0.809	0.374	−0.177		0.070	
***Model 3***															
*Cardiorespiratory fitness*															
20-m shuttle run test	0.309	0.002	0.949	0.402	−0.106	**0.008**	0.390		0.037	0.161	0.353	−0.026	0.364		
Relative VO_2_max	0.309	−0.001	0.964	0.402	−0.046	**0.008**	0.391		0.014	0.150	0.353	−0.010	0.360		
Absolute VO_2_max	0.317	0.000	0.344	0.340	0.000	0.454	0.398		0.000	0.087	0.349	0.000	0.538		
*Muscular fitness (MF)*															
Absolute upper-body MF	0.315	0.015	0.389	0.335	−0.007	0.743	0.389		0.013	0.177	0.362	−0.017	0.168		
Relative upper-body MF	0.310	−0.174	0.814	0.366	−1.292	0.073	0.375		0.255	0.715	0.366	−1.184	0.124		
Relative lower-body MF	0.332	0.004	0.095	0.335	0.001	0.774	0.380		−0.002	0.400	0.371	−0.004	0.087		
Absolute lower-body MF	0.341	0.000	0.051	0.344	0.000	0.337	0.374		0.000	0.894	0.361	0.000	0.187		
*Overall*															
Global MF	0.314	0.081	0.447	0.347	−0.115	0.267	0.375		−0.021	0.817	0.383	−0.230	**0.035**		
Overall fitness	0.311	0.050	0.655	0.383	−0.283	**0.025**	0.381		0.100	0.357	0.369	−0.175	0.100		

Model 1: analyses were controlled by age, educational centre and mother’s education level at follow-up, metabolic risk score 4 at baseline. Model 2: model 1 plus waist circumference at baseline. Model 3: model 1 plus body fat percentage at baseline. Statistically significant values are highlighted in bold

*Met4 *metabolic risk score excluding waist circumference, *VO*_*2*_*max *maximum oxygen consumption, *β *standardized coefficient

The longitudinal associations between BC variables at baseline and Met4 are depicted in Table 4. In male children, all BC variables (weight, BMI, %BF, and WC) were positively associated with Met4 (*β* = 0.017, *β* = 0.047, *β* = 0.011, *β* = 0.018, *p* < 0.020, respectively). Similar results were found in model 2 (*β* = 0.017, *β* = 0.047, *β* = 0.011, *β* = 0.017, *p* < 0.035, respectively), in model 3 (*β* = 0.018, *β* = 0.055, *β* = 0.014, *β* = 0.019, *p* < 0.035, respectively) and in model 4 (*β* = 0.017, *β* = 0.051, *β* = 0.013, *β* = 0.018, *p* < 0.038, respectively). In male adolescents, weight and BMI were positively associated with Met4 in model 1 (*β* = 0.011, *β* = 0.037, *p* = 0.04, *p* = 0.026, respectively), in model 2 (*β* = 0.013, *β* = 0.046, *p* = 0.025, *p* = 0.011, respectively), and in model 4 (*β* = 0.012, *β* = 0.045, *p* = 0.049, *p* = 0.024, respectively). Moreover, %BF was positively associated with Met4 in model 2 and model 4 (*β* = 0.013, *p* = 0.019, *p* = 0.044, respectively). Finally, in female children and adolescents not associations were found between BC variables and Met4 score (*p* > 0.05).

**Table 4:** Longitudinal association between body composition with Met4 in children and adolescents

	**Children (*n* = 188)**						**Adolescents (*n* = 195)**					
	**Male (*n* = 102)**			**Female (*n* = 86)**			**Male (*n* = 98)**			**Female (*n* = 97)**		
	Adjusted R^2^	*β*	*p*	Adjusted R^2^	*β*	*p*	Adjusted R^2^	*β*	*p*	Adjusted R^2^	*β*	*p*
***Model 1***												
Weight	0.318	0.017	**0.018**	0.359	0.008	0.222	0.387	0.011	**0.040**	0.342	0.003	0.575
Body mass index	0.334	0.047	**0.006**	0.365	0.025	0.141	0.393	0.037	**0.026**	0.345	0.013	0.443
Body fat percentage	0.317	0.011	**0.019**	0.344	0.001	0.895	0.382	0.009	0.057	0.354	0.009	0.187
Waist circumference	0.321	0.018	**0.015**	0.362	0.011	0.173	0.375	0.012	0.103	0.355	0.012	0.182
***Model 2***												
Weight	0.311	0.017	**0.031**	0.383	0.002	0.763	0.387	0.013	**0.025**	0.346	0.003	0.658
Body mass index	0.326	0.047	**0.011**	0.384	0.009	0.657	0.397	0.046	**0.011**	0.348	0.010	0.548
Body fat percentage	0.309	0.011	**0.034**	0.402	−0.011	0.142	0.390	0.013	**0.019**	0.353	0.007	0.317
Waist circumference	0.313	0.017	**0.026**	0.385	0.004	0.599	0.374	0.015	0.063	0.357	0.011	0.218
***Model 3***												
Weight	0.310	0.018	**0.033**	0.351	0.007	0.402	0.383	0.010	0.094	0.384	−0.001	0.810
Body mass index	0.330	0.055	**0.008**	0.356	0.023	0.268	0.386	0.035	0.077	0.384	−0.003	0.862
Body fat percentage	0.314	0.014	**0.025**	0.347	−0.004	0.581	0.375	0.008	0.180	0.383	0.001	0.925
Waist circumference	0.314	0.019	**0.026**	0.354	0.009	0.310	0.371	0.010	0.254	0.386	0.005	0.579
***Model 4***												
Weight	0.310	0.017	**0.038**	0.366	0.003	0.727	0.380	0.012	**0.049**	0.367	0.001	0.914
Body mass index	0.327	0.051	**0.011**	0.368	0.011	0.587	0.390	0.045	**0.024**	0.367	0.004	0.835
Body fat percentage	0.311	0.013	**0.036**	0.383	−0.012	0.166	0.381	0.013	**0.044**	0.369	0.004	0.637
Waist circumference	0.313	0.018	**0.032**	0.368	0.005	0.565	0.367	0.013	0.128	0.374	0.008	0.363

Model 1: Analyses were controlled by age, educational centre and mother´s education level at follow-up, metabolic risk score 4 at baseline. Model 2: model 1 plus cardiorespiratory fitness (paliers) at baseline. Model 3: model 1 plus global muscular fitness at baseline. Model 4: model 1 plus overall fitness score. Statistically significant values are highlighted in bold

Met4 metabolic risk score excluding waist circumference, β standardized coefficient

Table 5 shows the association between changes in PF variables and Met4 at follow-up and its changes. Changes in absolute CRF were longitudinally positively associated with Met4 only when %BF was included as a covariable in the model. This association was also observed when changes in Met4 levels were used as the dependent variable. Changes in absolute lower-body MF were positively associated with follow-up Met4 in male children when adjusted by WC and %BF (*β* = 0.000, *p* = 0.037, *p* = 0.031, respectively). In addition, changes in absolute upper-body MF were positively associated with follow-up Met4 in female children (*β* = 0.06, *p* = 0.003). No associations were found between changes in PF variables and follow-up Met4 in male and female adolescents as well as between changes in PF variables and changes in Met4 in both sexes and age groups (*p* > 0.05).

**Table 5:** Association between changes in fitness variables and Met4 at follow-up and its changes

	**Children (*n* = 188)**						**Adolescents (*n* = 195)**					
	**Male (*n* = 102)**			**Female (*n* = 86)**			**Male (*n* = 98)**			**Female (*n* = 97)**		
	Adjusted R^2^	*β*	*p*	Adjusted R^2^	*β*	*p*	Adjusted R^2^	*β*	*p*	Adjusted R^2^	*β*	*p*
**Follow-up**												
*Cardiorespiratory fitness (adj WC)*												
20-m shuttle run test	0.291	0.005	0.882	0.459	0.012	0.747	0.362	0.027	0.277	0.418	0.017	0.622
Relative VO_2_max	0.291	0.002	0.886	0.458	0.001	0.952	0.364	0.010	0.243	0.418	0.005	0.675
Absolute VO_2_max	0.314	0.001	0.096	0.474	0.000	0.162	0.382	0.000	0.061	0.417	0.000	0.739
*Muscular fitness (MF) (adj WC)*												
Absolute upper-body MF	0.291	0.003	0.909	0.484	0.039	0.070	0.363	0.011	0.276	0.427	0.018	0.248
Relative upper-body MF	0.296	−0.627	0.413	0.463	0.661	0.438	0.356	0.405	0.541	0.421	0.560	0.445
Relative lower-body MF	0.292	0.001	0.668	0.459	−0.001	0.735	0.353	0.000	0.926	0.417	−0.001	0.863
Absolute lower-body MF	0.327	0.000	**0.037**	0.468	0.000	0.257	0.358	0.000	0.426	0.416	0.000	0.980
*Global MF (adj WC)*	0.292	−0.046	0.705	0.459	0.047	0.716	0.354	0.045	0.633	0.420	0.087	0.529
*Overall fitness (adj WC)*	0.291	−0.011	0.930	0.460	0.092	0.598	0.361	0.098	0.303	0.420	0.093	0.496
*Cardiorespiratory fitness (adj %BF)*												
20-m shuttle run test	0.267	−0.008	0.825	0.425	−0.003	0.931	0.350	0.013	0.622	0.332	0.010	0.779
Relative VO_2_max	0.267	−0.004	0.786	0.426	−0.006	0.724	0.351	0.006	0.550	0.331	0.003	0.830
Absolute VO_2_max	0.308	0.001	**0.030**	0.477	0.001	**0.013**	0.378	0.000	0.055	0.351	0.000	0.126
*Muscular fitness (MF) (adj %BF)*												
Absolute upper-body MF	0.267	0.005	0.834	0.498	0.060	**0.003**	0.356	0.011	0.308	0.350	0.025	0.141
Relative upper-body MF	0.278	−0.872	0.257	0.433	0.863	0.338	0.348	−0.012	0.985	0.332	−0.207	0.792
Relative lower-body MF	0.266	0.000	0.962	0.429	−0.002	0.486	0.348	−0.001	0.802	0.331	0.000	0.932
Absolute lower-body MF	0.307	0.000	**0.031**	0.456	0.000	0.057	0.356	0.000	0.329	0.348	0.000	0.167
*Global MF (adj %BF)*	0.273	−0.106	0.375	0.426	0.033	0.803	0.348	−0.015	0.877	0.332	−0.044	0.772
*Overall fitness (adj %BF)*	0.270	−0.085	0.516	0.425	0.016	0.927	0.348	0.027	0.786	0.331	0.011	0.944
**Changes**												
*Cardiorespiratory fitness (adj WC)*												
20-m shuttle run test	0.174	0.014	0.713	0.219	0.029	0.486	0.124	0.032	0.241	0.219	0.013	0.714
Relative VO_2_max	0.175	0.008	0.606	0.217	0.009	0.619	0.129	0.013	0.177	0.219	0.004	0.737
Absolute VO_2_max	0.195	0.001	0.127	0.227	0.000	0.285	0.172	0.000	**0.016**	0.221	0.000	0.570
*Muscular fitness (MF) (adj WC)*												
Absolute upper-body MF	0.173	0.006	0.815	0.218	0.014	0.552	0.116	0.009	0.418	0.239	0.024	0.150
Relative upper-body MF	0.173	−0.153	0.866	0.214	0.177	0.851	0.108	0.088	0.903	0.226	0.694	0.372
Relative lower-body MF	0.172	0.000	0.986	0.214	0.000	0.969	0.109	0.000	0.840	0.218	−0.001	0.859
Absolute lower-body MF	0.176	0.000	0.533	0.215	0.000	0.721	0.116	0.000	0.410	0.218	0.000	0.930
*Global MF (adj WC)*	0.173	−0.021	0.886	0.214	0.017	0.907	0.109	0.020	0.843	0.223	0.110	0.455
*Overall fitness (adj WC)*	0.173	0.030	0.843	0.219	0.134	0.491	0.119	0.100	0.342	0.222	0.093	0.519
*Cardiorespiratory fitness (adj %BF)*												
20-m shuttle run test	0.164	0.001	0.979	0.155	0.014	0.746	0.107	0.029	0.309	0.101	0.009	0.818
Relative VO_2_max	0.164	0.003	0.883	0.154	0.002	0.901	0.112	0.012	0.225	0.101	0.003	0.837
Absolute VO_2_max	0.194	0.001	0.081	0.216	0.001	**0.024**	0.172	0.000	**0.009**	0.135	0.000	0.086
*Muscular fitness (MF) (adj %BF)*												
Absolute upper-body MF	0.165	0.007	0.775	0.190	0.038	0.087	0.105	0.011	0.344	0.139	0.032	0.068
Relative upper-body MF	0.166	−0.380	0.673	0.154	0.249	0.804	0.095	−0.085	0.909	0.101	0.011	0.990
Relative lower-body MF	0.165	−0.001	0.745	0.155	−0.001	0.688	0.095	0.000	0.999	0.101	0.000	0.979
Absolute lower-body MF	0.169	0.000	0.486	0.170	0.000	0.247	0.106	0.000	0.326	0.124	0.000	0.153
*Global MF (adj %BF)*	0.167	−0.072	0.605	0.153	−0.011	0.941	0.095	−0.008	0.939	0.101	0.000	1.000
*Overall fitness (adj %BF)*	0.165	−0.041	0.790	0.154	0.046	0.818	0.100	0.075	0.489	0.101	0.028	0.861

Association between changes in fitness indicators and changes in metabolic risk score 4 were adjusted by age, educational centre and mother´s education level at follow-up and changes in body composition variables. Statistically significant values are highlighted in bold

adj adjusted by, %BF body fat percentage, MF muscular fitness, Met4 metabolic risk score excluding waist circumference, VO_2_max maximum oxygen consumption, WC waist circumference, β standardized coefficient. Longitudinal analyses (when using follow-up values of the dependent variable) were controlled by age, educational centre and mother´s education level at follow-up, metabolic risk score 4 at baseline and changes in body composition variables

The associations between changes in BC variables and Met4 at follow-up and its changes are depicted in Table 6. Changes in body weight and BMI were positively associated with follow-up Met4 in male children when adjusted by CRF (*β* = 0.045 and *β* = 0.114, respectively, both *p* < 0.01); by MF (*β* = 0.044 and *β* = 0.104, respectively, both *p* < 0.01); and by overall fitness (*β* = 0.045 and *β* = 0.116, respectively, both *p* < 0.01) at follow-up. Moreover, changes in BMI were positively associated with changes in Met4 when adjusted by CRF, MF and overall fitness (*β* = 0.104, *β* = 0.093, *β* = 0.109, *p* = 0.016, *p* = 0.032, *p* = 0.017, respectively). All changes in BC variables were positively associated with Met4 at follow-up and changes in Met4 in female children, even when analyses were adjusted by changes in PF variables (all, *p* < 0.008). Changes in body weight were positively associated with changes in Met4 when adjusted by CRF and overall fitness in male adolescents (*β* = 0.023, *p* = 0.038, *p* = 0.044, respectively). All changes in BC variables were positively associated with Met4 at follow-up and changes in Met4 in female adolescents (except for %BF), even when we adjusted by changes in PF variables (all, *p* < 0.018).

**Table 6:** Association between changes in body composition variables and Met4 at follow-up and its changes

	**Children (*n* = 188)**						**Adolescents (*n* = 195)**					
	**Male (*n* = 102)**			**Female (*n* = 86)**			**Male (*n* = 98)**			**Female (*n* = 97)**		
	Adjusted R^2^	*β*	*p*	Adjusted R^2^	*β*	*p*	Adjusted R^2^	*β*	*p*	Adjusted R^2^	*β*	*p*
**Follow-up**												
*Body composition (adj CRF)*												
Weight	0.350	0.045	**0.001**	0.519	0.048	** < 0.001**	0.378	0.019	0.061	0.380	0.024	**0.018**
Body mass index	0.347	0.114	**0.002**	0.495	0.129	** < 0.001**	0.368	0.051	0.124	0.385	0.070	**0.012**
Body fat percentage	0.267	0.004	0.593	0.425	0.023	**0.002**	0.350	−0.003	0.740	0.332	0.000	0.952
Waist circumference	0.291	0.022	0.082	0.459	0.046	** < 0.001**	0.362	0.017	0.200	0.418	0.045	**0.001**
*Body composition (adj GMF)*												
Weight	0.349	0.044	**0.003**	0.523	0.050	** < 0.001**	0.369	0.017	0.085	0.383	0.027	**0.014**
Body mass index	0.341	0.104	**0.004**	0.503	0.141	** < 0.001**	0.358	0.042	0.208	0.391	0.083	**0.008**
Body fat percentage	0.273	0.003	0.700	0.426	0.024	**0.002**	0.348	−0.005	0.540	0.332	−0.002	0.852
Waist circumference	0.292	0.020	0.133	0.459	0.048	** < 0.001**	0.354	0.015	0.278	0.420	0.047	**0.001**
*Body composition (adj overall fitness)*												
Weight	0.350	0.045	**0.002**	0.523	0.050	** < 0.001**	0.375	0.019	0.064	0.382	0.025	**0.015**
Body mass index	0.345	0.116	**0.003**	0.503	0.139	** < 0.001**	0.367	0.053	0.120	0.388	0.075	**0.009**
Body fat percentage	0.270	0.003	0.761	0.425	0.023	**0.002**	0.348	−0.003	0.693	0.331	−0.001	0.938
Waist circumference	0.291	0.021	0.118	0.460	0.048	** < 0.001**	0.361	0.019	0.187	0.420	0.046	**0.001**
**Changes**												
*Body composition (adj CRF)*												
Weight	0.184	0.023	0.160	0.221	0.043	** < 0.001**	0.146	0.023	**0.038**	0.176	0.028	**0.009**
Body mass index	0.220	0.104	**0.016**	0.284	0.143	** < 0.001**	0.141	0.070	0.050	0.182	0.080	**0.006**
Body fat percentage	0.164	0.001	0.952	0.155	0.023	**0.005**	0.107	0.008	0.359	0.101	0.004	0.606
Waist circumference	0.174	0.015	0.327	0.219	0.050	** < 0.001**	0.124	0.023	0.126	0.219	0.049	**0.001**
*Body composition (adj GMF)*												
Weight	0.182	0.021	0.214	0.215	0.043	**0.001**	0.132	0.021	0.062	0.185	0.032	**0.005**
Body mass index	0.212	0.093	**0.032**	0.287	0.154	** < 0.001**	0.120	0.056	0.123	0.196	0.097	**0.003**
Body fat percentage	0.167	−0.001	0.901	0.153	0.023	**0.008**	0.095	0.004	0.629	0.101	0.004	0.643
Waist circumference	0.173	0.012	0.442	0.214	0.050	**0.001**	0.109	0.018	0.232	0.223	0.052	**0.001**
*Body composition (adj overall fitness)*												
Weight	0.183	0.023	0.179	0.221	0.045	** < 0.001**	0.139	0.023	**0.044**	0.179	0.029	**0.007**
Body mass index	0.220	0.109	**0.017**	0.296	0.155	** < 0.001**	0.134	0.071	0.056	0.187	0.086	**0.005**
Body fat percentage	0.165	−0.001	0.931	0.154	0.024	**0.005**	0.100	0.007	0.417	0.101	0.005	0.606
Waist circumference	0.173	0.014	0.371	0.219	0.052	** < 0.001**	0.119	0.023	0.134	0.222	0.050	**0.001**

Association between changes in body composition indicators and changes in metabolic risk score 4 were adjusted by age, educational centre and mother’s education level at follow-up and changes in fitness variables Statistically significant values are highlighted in bold

*adj *adjusted by, *CRF *cardiorespiratory fitness (paliers), *GMF *global muscular fitness, *Met4 *metabolic risk score excluding waist circumference, *β *standardized coefficient. Longitudinal analyses (when using follow-up values of the dependent variable) were controlled by age, educational centre and mother’s education level at follow-up, metabolic risk score 4 at baseline and changes in fitness variables

**Fig. 1:**
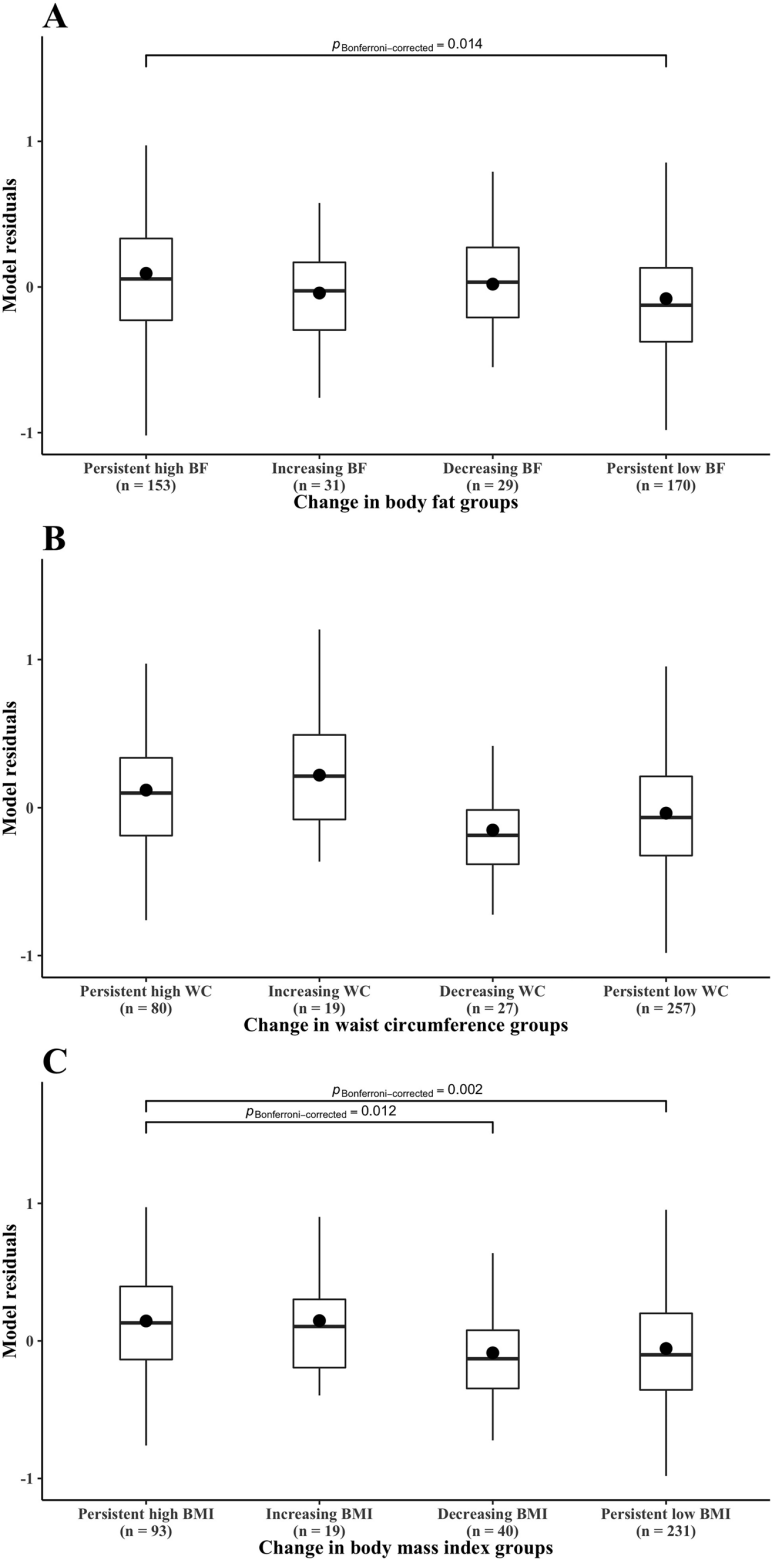
Differences in metabolic risk score 4 between changes in body compositions groups (**A**: body fat (%); **B**: waist circumference (cm); **C**: body mass index (kg/m^2^)). Dots indicated residuals mean of the lineal model were metabolic risk score 4 was dependent variable and age, educational centre, mother´s education level at follow-up and changes in fitness score were independent variables. The residuals median is indicated as horizontal bar included in the box plot. Only significant differences are shown at level P < 0.05 after Bonferroni´s correction

Differences in Met4 between changes in BC groups are displayed in Fig. 1. Significant differences were found between participants groups who were persistent high %BF and those who were persistent low %BF (mean = 0.09, mean =  −0.08, respectively, *p* = 0.014). Finally, significant differences were found between participants groups who were persistent high BMI and those who were persistent low BMI (mean = 0.14, mean =  −0.06, p = 0.002), and there were significant differences between participants groups who were persistent high BMI and those who were decreasing BMI (mean = 0.14, mean =  −0.09, p = 0.012).

## Discussion

Our results showed that most BC variables were longitudinally and positively associated with the Met4 in males children and adolescents. In contrast, some PF variables were negatively associated with Met4 mainly in females children and adolescents. Specifically, relative CRF was negatively associated in female children, and MF was negatively associated in female adolescents. In contrast, absolute CRF showed a positive association with Met4 in male children and adolescents. However, these associations were stronger for BC variables than for PF variables, even when the changes produced after 2 years were included as exposures. The lack of association between BC variables and Met4 in females could be explained by the few changes that occurred in these variables after a 2-year follow-up period. On the contrary, males suffered greater changes in BC variables than women. Therefore, from a statistical point of view, it is more likely to find a significant association when the independent variable has a greater range of values.

Regarding the association between PF and MetS, it is important to note that its direction and strength differ when using relativized or absolute PF variables. In regards to CRF, some authors have observed that when the VO2max is corrected by body size (i.e., body weight), the associations were negative, so that the greater the relative VO2max the lower the MetS. On the contrary, it would appear that this way of standardization coverts the positive correlation between the physiological performance variable and the body size variable into a negative one [42]. Our statistical analyses also showed differences in the direction of the association between CRF and Met4 when using absolute or relative CRF. In this sense, some authors suggest that the association between VO2 relativized by body weight (ml/kg/min) could be highly influenced by the association between body weight and health parameters, reflecting their level of fat more than their level of PF [43]. Besides, in terms of MF, previous evidence shows that when MF is relativized by body weight, there was a negatively association between MF and MetS [13, 20, 44–54]. On the contrary, when MF was considered in absolute terms, a positive association between MF and MetS has been observed in other studies [20, 21]. In this sense, we found different methods to evaluate MF. Most studies use the HG to assess upper body strength [20, 21, 44, 47–54] and the SLJ for lower body strength [21, 44, 49, 50, 54], and others used isometric dynamometers to assess different muscle groups [13, 45, 46]. To express the results of the HG, most divide the result by body weight [44, 48–51] or adjust the result by body weight [13, 20, 45, 46, 52–54], while few studies analysed absolute MF [20, 21]. Regarding the SLJ, most used the result obtained in centimetres [21, 44, 49, 54] while others multiplied it by body weight [50]. In the current study, we found opposite associations depending on whether we used MF in absolute or relative terms, observing a negative association when we used MF relativized by BC and a positive association when its absolute form was employed. The reason for this difference can be explained by the association between MF and some BC variables as adiposity [17]. For instance, in our sample, those children and adolescents who had higher body weight and higher BMI also had higher levels of absolute MF in the handgrip test and less distance in SLJ, being the latest a weight-bearing test (data not shown), in agreement with the results obtained by Artero et al. [55]. One possible explanation could be that participants with a higher weight level followed a diet that promoted a caloric surplus diet. A positive energy balance is associated with an increased body weight [56] but also with an increase in absolute muscle mass [57], which is a clear determinant of MF [14, 58]. For this reason, those children and adolescents with higher body weight had more muscle mass and obtained better results in the handgrip test. On the contrary, these young people obtained worse results in the SLJ since they had to mobilize all their body weight to perform this test. In this sense, our cross-sectional results showed how the different indicators of relative or absolute MF were negatively or positively associated with MetS 4, respectively. This may be because the relative variables include BC in the measure; therefore, this association between the relative PF and metabolic risk may be affected by the association between BC and metabolic risk factors [20].

In fact, we observed that BC measures were strongly and negatively associated with MetS, this result being explained because a substantial part of the pathophysiology of MetS is driven by the resistance to the metabolic effects of insulin. The major cause of insulin resistance in childhood is a typical lipid partitioning pattern characterized by increased deposition of lipids within insulin-responsive tissues, such as the liver and skeletal muscle and within the viscera [59]. This lipid deposition pattern is also associated with the infiltration of intra-abdominal tissues with cells of the immune system, inducing systemic, low-grade inflammation typically observed in insulin-resistant obese children and adolescents [59]. All these physiological processes lead to a deterioration of the cardiovascular system, which increases the probability of future cardiovascular events [60].

A previous review suggests that BC is more strongly associated with MetS than PF [61]. Furthermore, it is not only excess body weight at a given time point that is important, but also the progression or changes in this body weight status [62]. In this sense, a decrease in body weight produces a decrease in cardiometabolic risk [62], which may be because weight loss is associated with a decrease in inflammatory cytokines and insulin concentration and an increase in insulin sensitivity, improving metabolic health [63]. Conversely, an increase in weight leads to an increase in the incidence of cardiovascular events [63]. Additionally, given the relationship between PF and BC, both variables should be the focus of preventive strategies to reduce the incidence of MetS in children and adolescents. On the one hand, evidence indicates that increments in CRF will ameliorate the level of adiposity and, ultimately, the MetS risk [64, 65]. On the other hand, increased MF through resistance training promotes increased insulin sensitivity, which also affects the risk of MetS [19], a possible mechanism through which high MF may influence insulin resistance is by stimulating proteins in the insulin-signalling cascade [66]. Based on these results and considering the strong association between the BC variables and MetS, we consider that the CRF and MF variables related to BC are a better way to express the PF variables when the objective is to predict MetS. It is important to make a distinction between CRF and MF variables in absolute and relative terms in order to avoid terminological confusion and attribute benefits to MF that may come from the influence of body weight.

### Limitations and strengths

Several limitations should be mentioned. First, %BF was derived from an indirect measure of BC: skinfold thicknesses. The main disadvantage of using this technique is the need of expertise to carry out the measurements. Nonetheless, skinfold thicknesses were taken by trained professionals, and their validity has been previously established in the pediatric population [29]. Second, in the case of the 20-m shuttle run test, participants have to carry their weight over a series of 20-m shuttle runs, which adversely affects performance in the heavier participants [67]. Nonetheless, we used an equation to estimate CRF that was validated without correcting the test performance by any anthropometric measure [27]. Thus, the current results should be interpreted with caution, since no direct measure of absolute VO2 was available in our study. Third, since we used a longitudinal design, the causation of the associations could not be properly determined. Finally, the generalizability of these results should be cautiously considered cautiously because we could not determine the influence of ethnicity and country’s economic development on these associations, given that only urban, Caucasian Spanish children and adolescents participated in this study. Otherwise, the current research presents some strengths. The longitudinal design and the relatively large sample, which allows us to conduct the analyses differentiating by sex and age groups, are major strengths of the present study. Moreover, the use of clustered MetS risk factors has been suggested as a good indicator of cardiovascular health, compared with individual MetS risk factors [5].

### Conclusion

In conclusion, it seems that BC is more strongly associated with MetS and Met4 than PF in children and adolescents. Attaining/maintaining an optimal body weight status should be considered an important objective of health-promoting programs at both, childhood and adolescence, not forgetting to achieve appropriate PF levels. Furthermore, the way of expressing PF variables (absolute or relative) determines the direction of the association with MetS and Met4, and the existing negative association between these features when relative PF is employed. Taking all this information together, the relativized form of PF by BC seems a better screening tool as it appears to more fully represent the cardiometabolic health framework of young people than just including the absolute form of the PF.

## Abbreviations

BC: Body composition
%BF: Body fat percentage
BMI: Body mass index
CRF: Cardiorespiratory fitness
CVD: Cardiovascular disease
MetS: Metabolic syndrome score
Met4: Metabolic risk score excluding waist circumference
MF: Muscular fitness
PF: Physical fitness
SBP: Systolic blood pressure
SLJ: Standing long jump test
HDL-c: High-density lipoprotein cholesterol
HG: Handgrip strength
WC: Waist circumference
VO_2_max: Relative maximal oxygen uptake
